# Analysis and comparison of SARS-CoV-2 variant antibodies and neutralizing activity for 6 months after a booster mRNA vaccine in a healthcare worker population

**DOI:** 10.3389/fimmu.2023.1166261

**Published:** 2023-05-17

**Authors:** Sina Hosseinian, Rafael de Assis, Ghali Khalil, Madeleine K. Luu, Aarti Jain, Peter Horvath, Rie Nakajima, Anton M. Palma, Anthony Hoang, Eisa Razzak, Nicholas Garcia, Joshua Alger, Mina Kalantari, Emily K. Silzel, Algis Jasinskas, Frank Zaldivar, Sebastian D. Schubl, Philip L. Felgner, Saahir Khan

**Affiliations:** ^1^ School of Medicine, University of California Irvine, Irvine, CA, United States; ^2^ Department of Physiology and Biophysics, University of California Irvine, Irvine, CA, United States; ^3^ School of Biological Sciences, University of California Irvine, Irvine, CA, United States; ^4^ Institute for Clinical and Translational Science, University of California Irvine, Irvine, CA, United States; ^5^ Innovative Health Diagnostics, Irvine, CA, United States; ^6^ Department of Pediatrics, University of California Irvine, Irvine, CA, United States; ^7^ Department of Surgery, School of Medicine, University of California Irvine, Irvine, CA, United States; ^8^ Department of Medicine, Keck School of Medicine, University of Southern California, Los Angeles, CA, United States

**Keywords:** serology, vaccine, mRNA, healthcare worker (HCW), SARS-CoV-2

## Abstract

**Introduction:**

In the context of recurrent surges of SARS-CoV-2 infections, a detailed characterization of antibody persistence over a 6-month period following vaccine booster dose is necessary to crafting effective public health policies on repeat vaccination.

**Methods:**

To characterize the SARS-CoV-2 antibody profile of a healthcare worker population over a 6-month period following mRNA vaccination and booster dose. 323 healthcare workers at an academic medical center in Orange County, California who had completed primary vaccination and booster dose against SARS-CoV-2 were recruited for the study. A total of 690 blood specimens over a 6-month period were collected *via* finger-stick blood and analyzed for the presence of antibodies against 9 SARS-CoV-2 antigens using a coronavirus antigen microarray.

**Results:**

The primary outcome of this study was the average SARS-CoV-2 antibody level as measured using a novel coronavirus antigen microarray. Additional outcomes measured include levels of antibodies specific to SARS-CoV-2 variants including Delta, Omicron BA.1, and BA.2. We also measured SARS-CoV-2 neutralization capacity for a subset of the population to confirm correlation with antibody levels. Although antibodies against SARS-CoV-2 wane throughout the 6-month period following a booster dose, antibody levels remain higher than pre-boost levels. However, a booster dose of vaccine based on the original Wuhan strain generates approximately 3-fold lower antibody reactivity against Omicron variants BA.1 and BA.2 as compared to the vaccine strain. Despite waning antibody levels, neutralization activity against the vaccine strain is maintained throughout the 6-month period.

**Discussion:**

In the context of recurrent surges of SARS-CoV-2 infections, our data indicate that breakthrough infections are likely driven by novel variants with different antibody specificity and not by time since last dose of vaccination, indicating that development of vaccinations specific to these novel variants is necessary to prevent future surges of SARS-CoV-2 infections.

## Introduction

Since the initial 2019 outbreak of the novel beta coronavirus SARS-CoV-2, rapid international spread of the COVID-19 disease has resulted in a global pandemic. In efforts to contain the spread and severity of COVID-19, the FDA approved the emergency distribution of mRNA vaccines BNT162b2 and mRNA-1273 in December of 2020. Both vaccines provide high rates of protective efficacy of up to 95% against the targeted virus strain following two doses administered at least 3-4 weeks apart (1, 2). There has been a rapid global increase in SARS-CoV-2 cases since then, mainly due to the high infectivity and antibody escape mutations of the new Omicron variants, as well as waning immunity from the BNT162b2 and mRNA-1273 vaccines (3). The FDA has since approved the administration of additional “booster” doses of mRNA vaccines. A booster vaccine dose has previously been effective at protecting against severe COVID-19-related outcomes (4) and has been shown to substantially increase neutralizing antibodies (5, 6). The neutralizing ability of the antibodies has also differed among SARS-CoV-2 variants (7). Therefore, it is of great importance to elucidate the effectiveness of the booster vaccine in maintaining a persistent antibody response in a population consistently exposed to novel variants of SARS-CoV-2.

Here, we seek to analyze the initial rise and waning of SARS-CoV-2 antibody responses induced by the third-dose mRNA vaccine booster in a healthcare worker population over a 6-month period using a coronavirus antigen microarray, with direct comparison of antibodies against multiple variants of concern. Binding antibodies against SARS-CoV-2 antigens have been shown to correlate strongly with neutralizing antibodies, which are a critical component of clinical immunity (8–11). We confirm the correlation of measured antibody responses with SARS-CoV-2 neutralizing capacity for a subset of 30 healthcare workers utilizing an FDA-authorized surrogate neutralization assay (12, 13).

## Methods

### Study population

This study was approved by the institutional review board (IRB) of the University of California Irvine (UCI) prior to initiation of the study (protocol HS-20205818). Widespread mRNA vaccination of healthcare workers (HCWs) at UC Irvine Health began in December 2020, administering over 16,000 doses of mRNA-1273 (Moderna-NIAID) or the BNT162b2 (Pfizer-BioNTech) vaccines within the first 4 months. In September of 2021, the FDA approved and UCI secured and administered booster shots to all HCWs who wished to be additionally vaccinated. All HCWs working at the UCI Medical Center, located in Orange County, CA, who participated in our previous study (14) were invited to receive serological testing by providing serum blood samples *via* a fingerstick directly before vaccination, 1-2 weeks after vaccination, 2 months, 4 months, and 6 months after booster vaccination. All blood samples were brought to the Institute for Clinical and Translational Science Core Laboratory at the UCI Medical Center. Serum samples were centrifuged using the Eppendorf 5415R and spun at 3000xg for 5 minutes. Serum was quickly transferred into a clean sterile tube and frozen at -80°C until analyzed for IgGs. Reports of their serological test results were returned within 4 weeks of receiving the test. At each assessment, demographic and work-related characteristics, testing frequency, exposure risk, and symptom history were collected *via* surveys administered prior to serum sample collection. Longitudinal participation was encouraged through an aggressive email campaign as well as ensuring that participants received a report of their antibody levels, but not every subject participated at every time point. Subjects who participated provided written consent in the form of an electronic document that was physically signed using an iPad or cellular device.

### Coronavirus antigen microarray

953 independent finger stick blood serum samples were collected over the 6-month period for analysis. This analysis was restricted to 690 samples to adhere to the pre-specified guidelines (**Table 1**). Specimens were probed and analyzed on a coronavirus antigen microarray (CoVAM) for IgG and IgM antibodies against 37 antigens from SARS-CoV-2, other coronaviruses, and other respiratory viruses using a coronavirus antigen microarray. The CoVAM contained 10 SARS-CoV-2 antigens including nucleocapsid protein (NP) and several varying fragments of the spike (S) protein, as well as 4 SARS, 3 MERS, 12 Common CoV, and 8 influenza antigens. A full list of antigens used in the assay can be found in **Supplementary Figure 1**. Samples were tested in triplicate. For more detailed information regarding the coronavirus antigen microarray, analysis, refer to our previous work (14).

**Table 1:** Participant demographics.

	HCWs (n= 323)
Gender	
Female	240 (74.3%)
Male	65 (20.1%)
Non-binary	1 (0.3%)
Declined to respond	17 (5.3%)
Vaccine type	
BNT162b2	234 (72.4%)
mRNA-1273	51 (15.8%)
Declined to respond	38 (11.8%)
Age	
<55 years	256 (79.2%)
≥55 years	53 (16.4%)
Declined to respond	14 (4.3%)
Race	
Asian	84 (26.0%)
White	49 (15.2%)
Latino/Hispanic	33 (10.2%)
Black	1 (0.3%)
Pacific Islander	5 (1.5%)
Other	3 (0.9%)
Declined to respond	148 (45.8%)
Role	
Administrative	22 (6.8%)
Food/EVS	12 (3.7%)
Nurse	134 (41.5%)
Physician	35 (10.8%)
Student	9 (2.8%)
MA/technician	8 (2.5%)
Other	87 (26.9%)
Declined to respond	16 (5.0%)

In summary, the raw data were checked for quality and normalized using a composite of methods described by Duarte (2013) and Bolstad (2003). Then, using a collection of known samples (here referred as “training set”, known to be positive or negative for SARS-CoV-2), a prediction model was constructed using a logistic regression, in which a combination of SARS-CoV-2 antigens were used as well as a Random Forest model using the full SARS-CoV-2 antigen collection. For single antigen reactivity prediction, a logistic regression model was built for each individual antigen, also using the training set. As a surrogate marker for exposure (in contrast to mRNA vaccination), reactivity to the nucleoprotein, as determined by the Logistic regression model, was used.

This model was found to be 93% sensitive and 98% specific in correctly classifying 91 PCR-positive cases and 88 pre-pandemic negative control (15). The model was then used to generate a weighted composite measure of IgG reactivity across antigens, with weights corresponding to each antigen’s relative importance in the model. This composite IgG reactivity measure was scaled up to represent the weighted mean fluorescence intensity (MFI) of all antigens assayed in the CoVAM. Here, we utilized a model containing all SARS-CoV-2 antigens as above with the exception of NP, as this antigen was used to classify prior exposure to SARS-CoV-2 in a subgroup analysis.

### Statistical analysis

In order to characterize SARS-CoV-2 antibody response over time, we fit a third-degree polynomial model of the average IgG reactivity measure using all available data from n=323 HCWs. Due to the variability in the timing of the tests across individuals, we report the model-estimated average IgG reactivity means and standard error of the mean (SEM) error bars at pre-boost, 1-2 weeks, 2 months, 4 months and 6 months post-boost dose, and compared the changes over time. We then explored differences in long-term antibody response by individual characteristics hypothesized to influence the magnitude and durability of the vaccine-induced antibody response: history of PCR positivity [yes or no], presence of systemic side effects (side effects used in this study include fever, chills, cough, difficulty breathing, congestion, loss of smell or taste, new onset fatigue, myalgia other than site of injection, and headache [none, mild, moderate, or severe] exposure in community [yes or no], HCW role [patient care vs. non-patient care role, gender [male or female, by self-report], age [≥55 vs. <55 years, by self-report]. We tested each potential moderator individually by fitting the same linear mixed effect model with the inclusion of an interaction term between that variable and time (e.g., time * age≥55 vs. <55 years).

In order to determine the correlation between reactivity of SARS-CoV-2 antigens and reactivity of antigens from the other viruses in the array, a correlation matrix using the function rcorr() from the Hmisc (version 5.0-1) package. The correlation matrix was visualized using the corrplot (version 0.92) package. PCA analysis was performed using the tidyverse (version 1.3.2) and factoextra (version 1.0.7) packages. All analyses were conducted using R programing environment v4.1.1.

### Neutralization assay

In order to accurately assess neutralization capacity of SARS-CoV-2 antibodies, we utilized a FDA-authorized research use only surrogate virus neutralization test provided by GeneScript and carried out through a local commercial lab, Innovative Health Design lab located in Irvine, California. Samples and controls are pre-incubated with HRP bound RBD to allow for interaction and binding of neutralization antibodies to RBD-HRP. The mixture is subsequently added to a pre-coated plate of hACE2 protein. Unbound RBD-HRP or RBD-HRP bound to non-neutralizing antibody will be captured by the plate. Neutralization antibodies complexed to RBD-HRP remains in the supernatant and is removed through washout steps. The reaction is quenched and samples are read at 450 nm in a microtiter plate reader. Absorbance of the sample is inversely dependent on the titer of the neutralizing antibodies, numbers are shown as a percentage of controls. Sera of 30 individuals who participated at every timepoint were tested using this surrogate neutralization assay to assess neutralizing capacity for binding of spike RBD fragments from the original Wuhan strain to human ACE2 receptors.

### Data availability

The data that support the findings of this study are available from the corresponding author upon reasonable request.

## Results

### SARS-CoV-2 antibody levels 6 months post-vaccination

**Figure 1:**
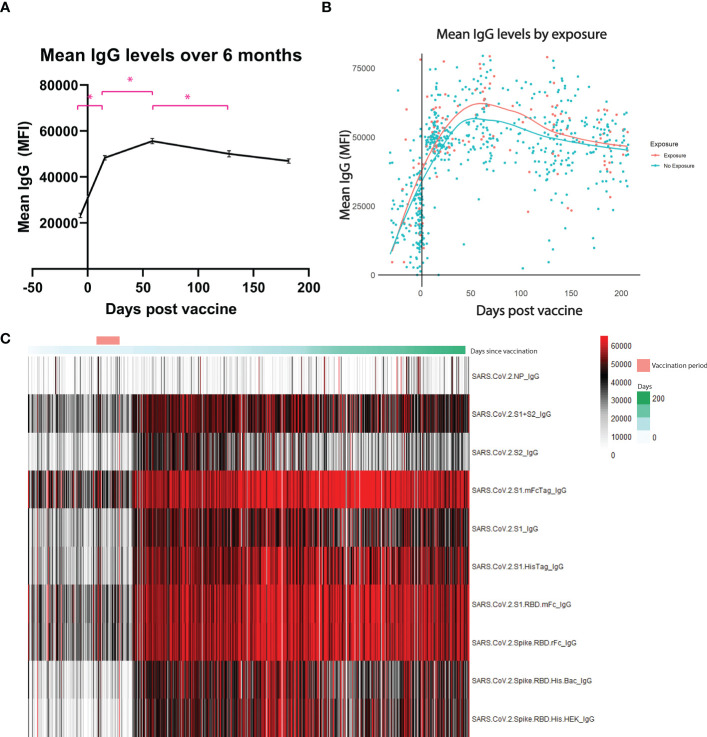
Antibody levels over 6 months. Mean IgG antibody levels measured by mean fluorescence intensity (MFI). **(A)** shows each individual sample collected over the 6-month period. **(B)** shows the difference between those who were exposed to the coronavirus at any point before the booster versus those who had no exposure. Exposure is deemed through presence of antibodies against the nucleocapsid protein, a protein only found in the virus and not in the vaccine. **(C)** is a heatmap of each antigen measured throughout the 6 months. * represents statistically significance, p < 0.05.

A total of 690 tests were analyzed from 323 HCWs throughout a 6-month period following booster vaccine dose (**Table 1**). Composite IgG antibody levels as measured by mean fluorescence intensity significantly increased in the 2 weeks following the booster vaccination (mean 23,303 vs 48,384, *p <*0.01) (**Figure 1**). Antibody levels continued to increase from 2 weeks post-boost until 2 months post-boost (mean 48,384 vs. 55,613, *p* < 0.01) but decreased significantly between months 2 and 4 (mean 55,613 vs. 50,105, *p* < 0.01) with a non-significant trend towards decreasing antibodies between months 4 and 6 (mean 50,105 vs 46,946, *p* = 0.14) Despite waning, antibody levels at 6 months post booster dose remain significantly higher than pre-boost levels.

To infer natural exposure, we assessed reactivity to the SARS-CoV-2 nucleocapsid protein, as this antigen is not present in currently approved SARS-CoV-2 vaccines. Exposure threshold was determined by logistic regression using the training set as reference. As seen on **Figure 1B**, no significant difference was observed between the groups at any of the timepoints tested.

### Antibody levels by demographic

**Figure 2:**
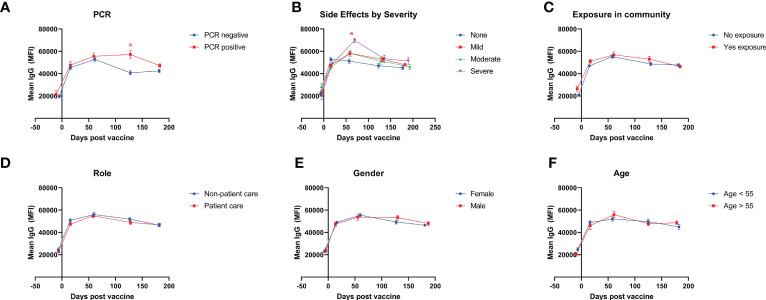
Antibody levels after vaccination for demographic subgroups. Mean IgG antibody levels measured throughout 6 months are compared for self-identified subgroups divided by **(A)** history of PCR-confirmed SARS-CoV-2 infection after booster vaccination, **(B)** severity of vaccine side-effects **(C)** exposure in community after booster vaccination, **(D)** healthcare worker role, **(E)** gender, and **(F)** age. * represents statistically significance, p < 0.05.

The cohort of HCWs were divided into subgroups based on their gender, age, presence of exposure in their community, worker role, presence of comorbidities, and history of PCR-confirmed SARS-CoV-2 infection. Individuals with a history of PCR-confirmed infection after their booster were found to have higher antibody levels at the 4 month timepoint (57,262 vs 40,846, *p* < 0.01) and at the 6 month timepoint (47,222 vs 42,451, *p* = 0.04) (**Figure 2A**). Vaccination systemic side effects were assessed through a 45-point subjective scale of severity encompassing 9 systemic side effects with subjective, self-reported severity from 0 to 5 points. Baseline antibody levels were equivalent between those with no systemic side effects and those with severe systemic side effects (21,639 vs 20,872, *p* = 0.85), but those with severe systemic side effects had significantly higher antibody levels at 2 months after booster vaccination (51,313 vs 69,849, *p* < 0.01), with a continued non-significant trend towards higher antibody levels compared to those without systemic side effects at later time points (**Figure 2B**). Stratification by community exposure, healthcare worker role, gender, and age did not yield any significant differences between groups (**Figures 2C–F**).

### Antibody levels against variants of concern

**Figure 3:**
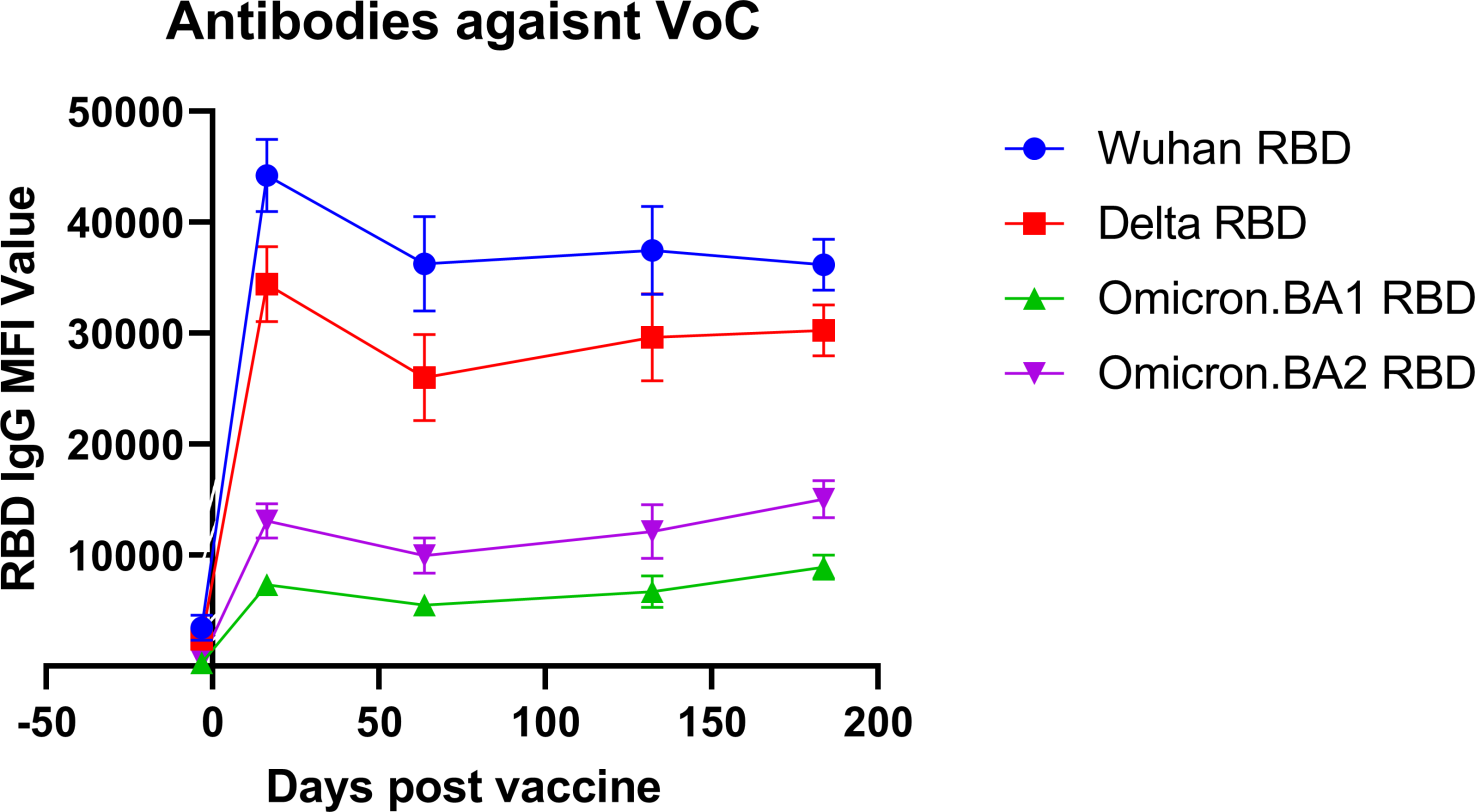
Antibody reactivity against variant RBD antigens. The antibody reactivity as measured by mean fluorescence intensity (MFI) against variant-specific receptor-binding domain (RBD) antigens is shown for different variants of concern (VoC) at different time points after vaccination.

Blood specimens from 30 healthcare workers were retrospectively chosen, based on specimen volume and availability at multiple time points and representativeness compared to the study population, to undergo further analysis using a more advanced microarray that included antigens against the original Wuhan strain and 3 variants of concern: Delta, Omicron BA.1, and BA.2. This analysis was restricted to analyze only the receptor-binding domain (RBD) for unbiased comparison between variants and included a total of 213 samples over the 6-month period, with a minimum of 27 samples per time point (**Figure 3**). Prior to booster vaccine dose, antibody levels against the Wuhan strain were not significantly different from the Delta strain (mean 3473 vs. 2449, *p* = 0.24) but were significantly lower against Omicron strains BA.1 and BA.2 (mean 3473 vs. 300, *p* < 0.01 and 3473 vs. 542, *p* < 0.01). At 2 weeks post booster vaccination, all variants had significantly lower antibody levels directed against them as compared to the Wuhan strain (Delta: 1.3 fold-reduction, *p <*0.01 Omicron BA.1 6.0 fold-reduction, *p* < 0.01; BA.2 3.4 fold reduction, *p* = <0.01). This trend persisted over the 6-month period.

### Antibody levels against other respiratory viruses

**Figure 4:**
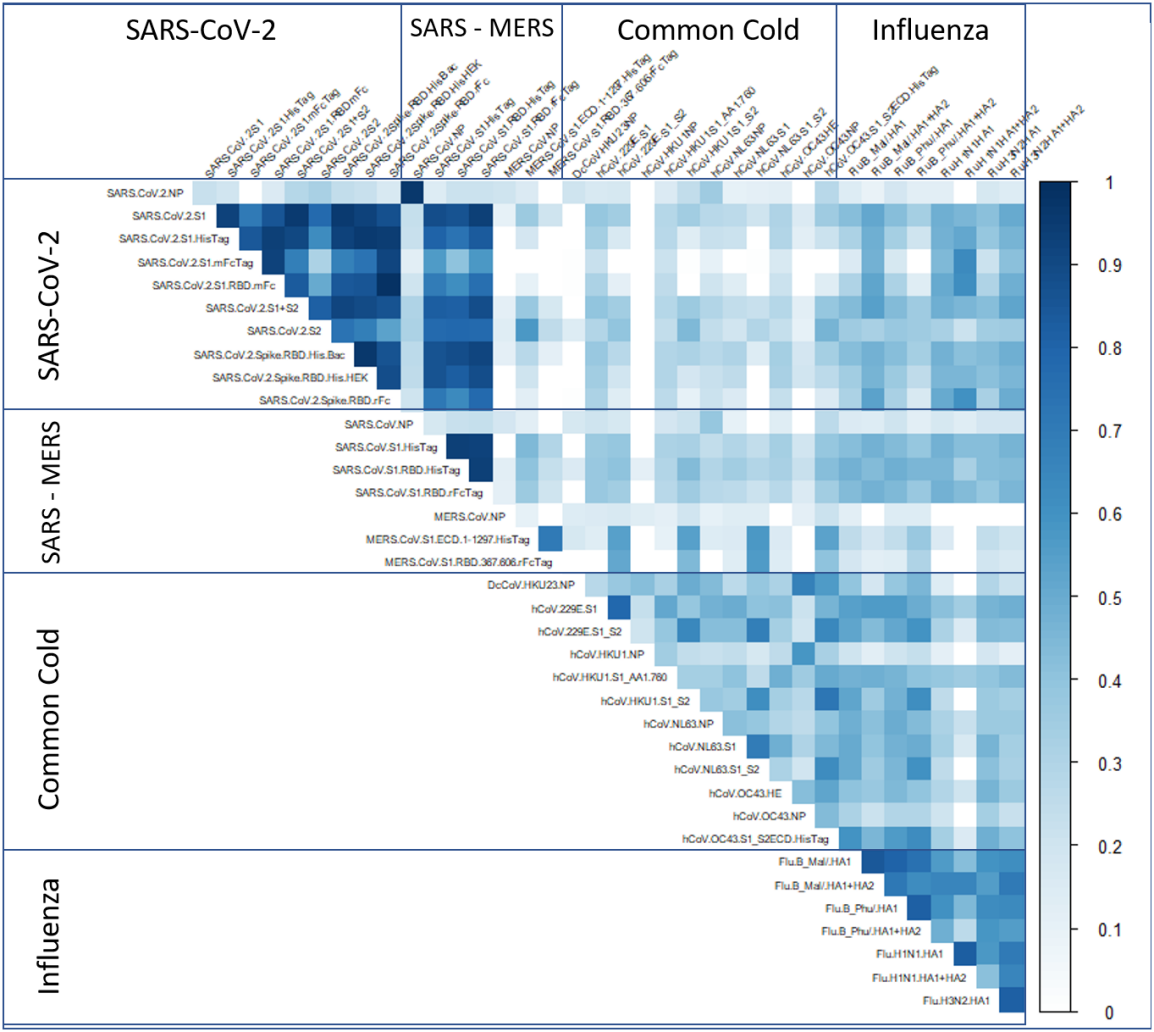
Correlation of antibodies against other respiratory viruses. Using COVAM 5, we are able to measure antibodies against SARS, MERS, CoV, and influenza in addition to SARS-CoV-2. This figure represents the correlation coefficient for each antibody against antibodies from other respiratory viruses.

Reactivity to antigens from all viruses represented on the array was observed for multiple samples. However, no significant correlation between the reactivity to SARS-CoV-2 antigens and either endemic coronavirus (229E, NL63, OC43, HKU1) or influenza virus antigens was observed. In accordance with our previous observations and the literature, this can be explained by a low cross reactivity between SARS-CoV-2 S1 and RBD antigens and the same antigens on other coronaviruses. Nevertheless, as shown on **Figure 4**, the observed correlation between different fragments of SARS-CoV-2 spike antigen was high (averaging r > 0.8) except for the nucleoprotein (averaging a r < 0.3). This observation confirms that the microarray can distinguish between antibody responses induced by vaccines that include spike protein antigens and natural infection which includes exposure to both spike and nucleocapsid protein antigens.

### Persistence of neutralizing antibodies against Wuhan strain despite waning antibody levels

The same 30 healthcare workers who were selected to undergo variant testing were also selected for analysis of neutralization capacity. These 30 individuals expressed a similar trend to the overall cohort, with their average antibody levels initially increasing from pre-boost to 2 months post-boost, but significant waning between months 2 to 6. Signal inhibition of neutralizing titers increased from 77% before booster vaccination to 96% immediately after booster vaccination (*p* < 0.01). Despite waning antibody levels from months 2 to 6, signal inhibition did not wane significantly over this time (**Supplementary Figure 2**).

## Discussion

In summary, we report data on the boosting of both composite IgG antibody levels and antibody neutralizing activity by a third dose of mRNA vaccine against SARS-CoV-2. The strengths of our study include a 6-month follow-up period, the comparison of antibody responses against SARS-CoV-2 variants of concern, and inclusion of subgroup analyses with respect to demographics, vaccine side-effects, and breakthrough SARS-CoV-2 infection. The weaknesses of our study include limited power to detect differences in subgroups and limitations of the HCW population, which is a relatively young and healthy population with few individuals who are immunocompromised or over the age of 65.

Both IgG antibody levels and neutralization activity have been shown to wane over the course of months following vaccination (14, 16). The booster vaccine dose has been shown to significantly increase both IgG antibody levels and neutralization activity (5, 17). Similarly, the booster vaccine dose has been shown to reduce risk of SARS-CoV-2 breakthrough infections (6). However, previous studies have focused on short-term effect of booster vaccination on SARS-CoV-2 antibody responses (18), while we chose to observe the study population over a longer 6-month follow-up period.

Using our CoVAM assay, we observed a significant increase in antibody levels following the booster dose of the vaccine. Despite significant waning between months 2-6, the antibody levels at 6 months remain higher than pre-boost levels. We also show no significant difference between individuals who were exposed to SARS-CoV-2 (as determined through reactivity to the nucleocapsid protein) compared to those who had no previous exposure. This data is limited in the ability to detect a small effect of previous exposure by our small sample size of individuals with previous exposure. Others have shown longer-lasting immunity as exhibited by higher antibody levels and neutralization activity in those with previous infection 9 months after primary 2-dose COVID-19 mRNA vaccination (19). In the context of the high rate of breakthrough infections prior to 6 months observed in recent surges of SARS-CoV-2 infections, these data indicate that waning of antibody levels over time is not the primary driver of SARS-CoV-2 breakthrough infections.

We stratified study participants based on gender, age, community exposure, worker role, and breakthrough COVID-19 infection based on a history of PCR positivity. We did not find significant differences in antibody levels for subgroups stratified by gender, age, self-reported community exposure to COVID-19, or clinical role, although the study may have been underpowered to detect small differences within these subgroups. We had a small cohort of individuals who had breakthrough infections confirmed through PCR positivity, and these individuals showed less waning in antibody responses with significantly higher antibody levels at 4 and 6 months.

We, among others, hypothesized that the quantity and severity of systemic side effects may be correlated with a more robust immune response, resulting in higher antibody titers. We show that the presence of systemic side effects is associated with higher antibody titers up to 4 months following booster vaccination. Severe systemic side effects are associated with higher antibody levels in a longitudinal fashion throughout the 6-months period. These data can reassure patients that systemic side-effects associated with vaccination may represent a more robust immune response to the vaccine dose.

The neutralizing capacity of SARS-CoV-2 antibodies has been shown to increase quickly following the vaccine booster dose (20). While we focused on binding antibody levels in this study, we did confirm correlation between binding antibodies and neutralizing antibodies in a subset of patients using an FDA-authorized surrogate neutralization assay (12). We retroactively selected 30 individuals who participated at every time point available, including before booster vaccination, in order to get a complete timeline of neutralization throughout the 6 month period. We acknowledge that this may lead to some selection bias, as those who come to every collection offered may be more likely to be more compliant with masking and exposure guidelines. We report significant increase in neutralizing antibodies against the original Wuhan strain following booster vaccine dose with maintenance throughout the 6-month period in this cohort, despite waning binding antibody levels measured on the CoVAM. It is important to note that our neutralization data are limited in that our assay was likely saturated at post-boost time points, with our values being at 96% inhibition, which may not fully allow us to detect small differences between post-boost timepoints. However, based on these data, we hypothesize that the decrease in antibody levels over the course of the 6-month period is not sufficient to reduce neutralization capacity and therefore unlikely clinically significant given correlation between neutralization capacity and clinical immunity.

We acknowledge that others have directly compared the neutralization capacity of vaccinee sera against SARS-CoV-2 variants of concern (20), however, estimates for the magnitude of antibody escape by the Omicron variant of concern vary widely from as low as 1.5-fold to as high as 32-fold (21) (22) (23, 24). Using an updated version of the CoVAM, we were able to directly compare antibodies elicited by booster vaccination against the spike protein receptor-binding domain antigens of multiple variants of concern. Our data show that antibodies elicited by booster vaccination directed against the original Wuhan strain show only a small non-significant decrease in binding to RBD antigen from the Delta variant but show significant decreases in binding to Omicron variants BA.1 (4-fold decrease) and BA.2 (3-fold decrease). Although the booster dose is effective in maintaining antibodies against Wuhan and Delta strain antigens, these data suggest that vaccines directed against Omicron strain antigens are needed to effectively prevent breakthrough COVID-19 infections.

## Data availability statement

The raw data supporting the conclusions of this article will be made available by the authors, without undue reservation.

## Ethics statement

The studies involving human participants were reviewed and approved by UCI IRB. The patients/participants provided their written informed consent to participate in this study.

## Author contributions

SH, GK, ML, AP, SS, PF, and SK conceived and designed research. SH, GK, ML, AH, ES, and NG collected samples. PH, JA, and FZ prepared and stored samples. PF and SK designed the microarray. RA, AJ, RN, and AJ constructed the microarray and probed samples. RA analyzed data. SH, GK, SS, PF, and SK interpreted results of data. SH and RA prepared figures. SH, GK, ML, NG, and SK drafted the manuscript. SH and SK edited and revised the manuscript. SH, SS, PF, and SK obtained funding for the project. All authors contributed to the article and approved the submitted version.

## Supplementary material

Supplementary Figure 1 Antibodies tested through COVAM 5.

Supplementary Figure 2 Neutralizing capacity of vaccinee sera. Neutralizing capacity of sera, measured as percent signal inhibition of binding of RBD antigen from the Wuhan strain to human ACE2 receptors compared to negative control sera, is shown for different time points after vaccination.
