# Supplementary figures and images for: Analysis and comparison of SARS-CoV-2 variant antibodies and neutralizing activity for 6 months after a booster mRNA vaccine in a healthcare worker population
Source: Front Immunol. 2023 May 17;14:1166261. doi: 10.3389/fimmu.2023.1166261 (PMC10229859; doi:10.3389/fimmu.2023.1166261)

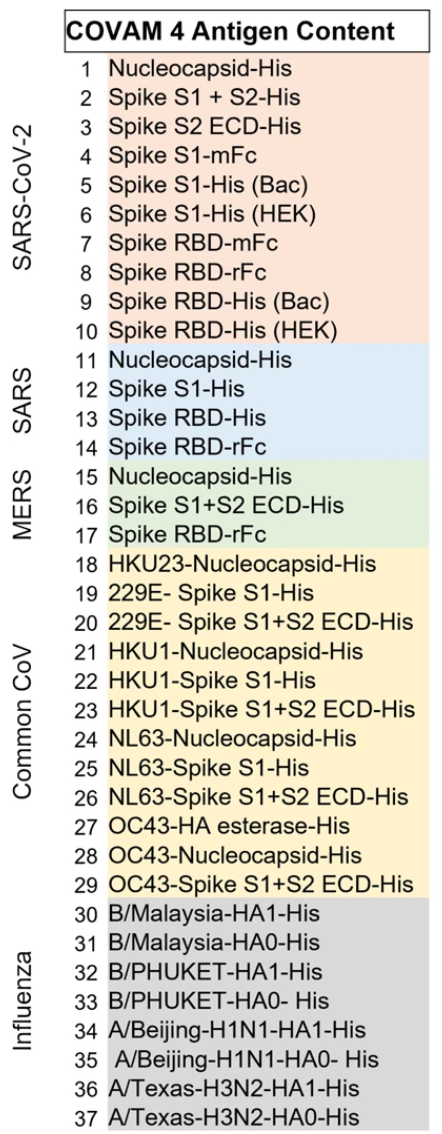

Supplement: Supplementary Figure 1 — Antibodies tested through COVAM 5. [file Image_1.png]

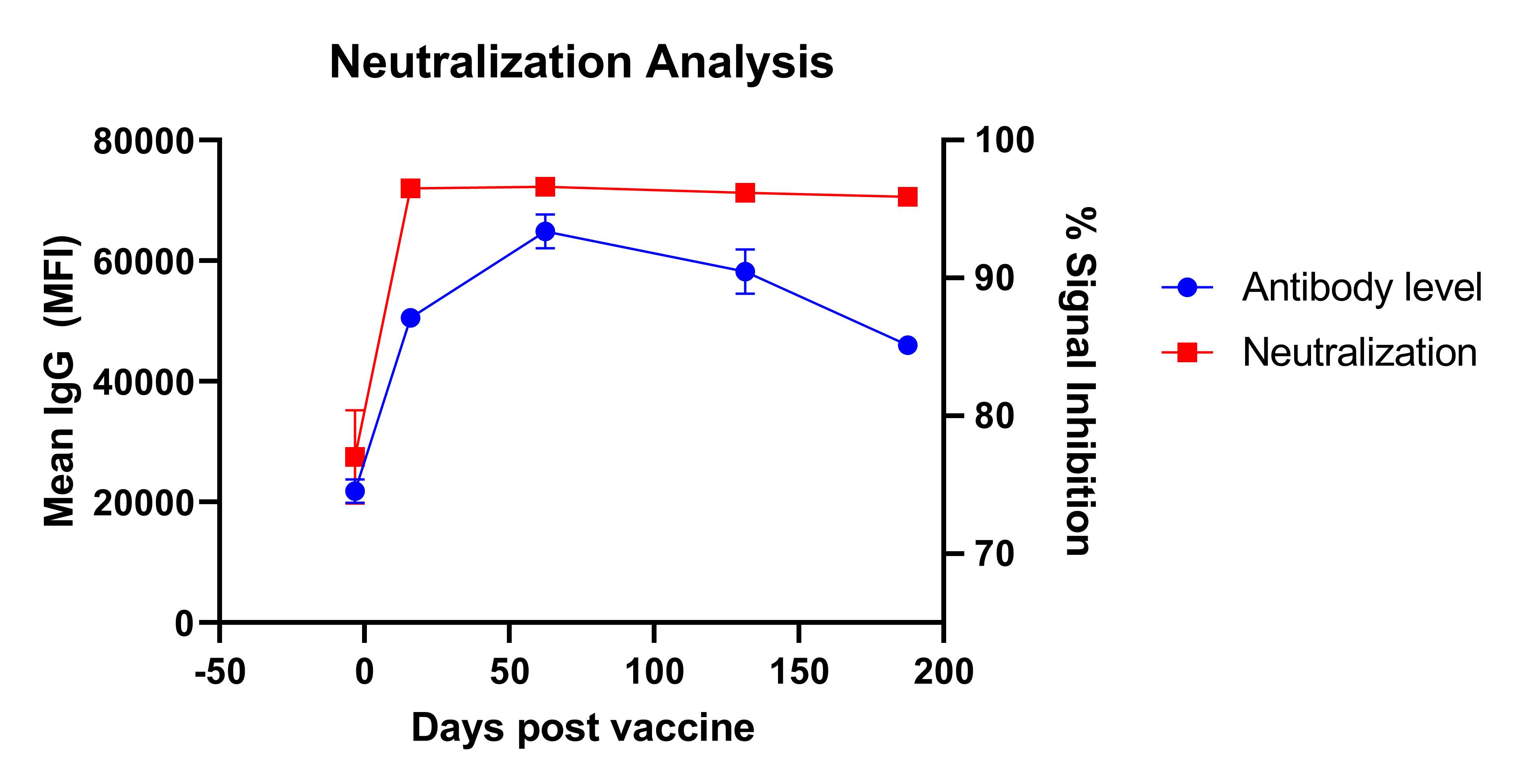

Supplement: Supplementary Figure 2 — Neutralizing capacity of vaccinee sera. Neutralizing capacity of sera, measured as percent signal inhibition of binding of RBD antigen from the Wuhan strain to human ACE2 receptors compared to negative control sera, is shown for different time points after vaccination. [file Image_2.tif]
